# *Arabidopsis* Heat Stress-Induced Proteins Are Enriched in Electrostatically Charged Amino Acids and Intrinsically Disordered Regions

**DOI:** 10.3390/ijms19082276

**Published:** 2018-08-03

**Authors:** David Alvarez-Ponce, Mario X. Ruiz-González, Francisco Vera-Sirera, Felix Feyertag, Miguel A. Perez-Amador, Mario A. Fares

**Affiliations:** 1 Biology Department, University of Nevada, Reno, NV 89557, USA; ffeyertag@unr.edu; 2 Instituto de Biología Molecular y Celular de Plantas, CSIC-UPV, 46022 Valencia, Spain; marioxruizgonzalez@gmail.com (M.X.R.-G.); fravesi@ibmcp.upv.es (F.V.-S.); mpereza@ibmcp.upv.es (M.A.P.-A.); 3 Smurfit Institute of Genetics, University of Dublin, Trinity College, Dublin 2, Ireland

**Keywords:** temperature response, protein thermostability, salt bridges, intrinsically disordered proteins

## Abstract

Comparison of the proteins of thermophilic, mesophilic, and psychrophilic prokaryotes has revealed several features characteristic to proteins adapted to high temperatures, which increase their thermostability. These characteristics include a profusion of disulfide bonds, salt bridges, hydrogen bonds, and hydrophobic interactions, and a depletion in intrinsically disordered regions. It is unclear, however, whether such differences can also be observed in eukaryotic proteins or when comparing proteins that are adapted to temperatures that are more subtly different. When an organism is exposed to high temperatures, a subset of its proteins is overexpressed (heat-induced proteins), whereas others are either repressed (heat-repressed proteins) or remain unaffected. Here, we determine the expression levels of all genes in the eukaryotic model system *Arabidopsis thaliana* at 22 and 37 °C, and compare both the amino acid compositions and levels of intrinsic disorder of heat-induced and heat-repressed proteins. We show that, compared to heat-repressed proteins, heat-induced proteins are enriched in electrostatically charged amino acids and depleted in polar amino acids, mirroring thermophile proteins. However, in contrast with thermophile proteins, heat-induced proteins are enriched in intrinsically disordered regions, and depleted in hydrophobic amino acids. Our results indicate that temperature adaptation at the level of amino acid composition and intrinsic disorder can be observed not only in proteins of thermophilic organisms, but also in eukaryotic heat-induced proteins; the underlying adaptation pathways, however, are similar but not the same.

## 1. Introduction

Proteins of thermophilic prokaryotes (those adapted to high temperatures) exhibit several distinctive features that increase their thermostability. One of the most consistent observations in thermophile proteins is an enrichment in salt bridges [1,2]. Salt bridges consist of electrostatic interactions among amino acid residues with positive (Lys and Arg) and negative (Glu and Asp) charges, and their contribution to increasing the stability of thermophilic bacteria was first proposed by Perutz and Raidt [3]. In addition, compared with proteins of mesophiles (adapted to intermediate temperatures) and psychrophiles (adapted to low temperatures), thermophile proteins tend to exhibit more disulfide bonds and non-covalent interactions, including hydrogen bonds, and hydrophobic interactions, features that also tend to increase protein stability by linking together distant parts of the amino acid sequence [4,5]. These structural trends have an impact on the amino acid composition of thermophilic proteomes: the proteins of thermophilic bacteria tend to be enriched in charged amino acids and depleted in polar ones such as Ser, Thr, Asn, and Gln [6,7,8,9,10,11,12].

A few studies in prokaryotes have also shown that thermophile proteins are depleted in intrinsically disordered regions (IDRs), i.e., regions that lack a defined three-dimensional structure [13,14,15]. This observation is consistent with the fact that high temperatures induce disorder, but in contrast with the fact that IDRs confer thermoresistance [16,17,18].

Much less is known about how eukaryotic proteomes adapt to high temperatures. Some studies have suggested that the same biases in amino acid composition observed in thermophilic prokaryotes can be observed in thermophilic fungi (compared to other fungi; ref. [19]) and endothermic vertebrates (compared to ectothermic vertebrates; ref. [20]). In agreement with this notion, comparison of the orthologous proteins of two closely related fish, *Pachycara brachycephalum* (from Antarctica) and *Zoarces viviparous* (from a temperate zone) revealed an excess of Ser and a reduction of Glu and Asn in the cold-adapted species [21]. To our knowledge, the relationship between temperature and intrinsic disorder has not been investigated in eukaryotic proteomes.

Protein adaptation to high temperatures is expected to be observed not only in the proteins of thermophilic organisms, but also in some of the proteins of any mesophilic organism. When an organism is exposed to high temperatures, a subset of its proteins is overexpressed, whereas others are repressed (heat-induced and heat-repressed proteins, respectively, e.g., ref. [22]). As heat-induced function at relatively high temperatures, we hypothesize that they should be similar to those of thermophilic organisms.

Plants represent particularly suitable models to test this hypothesis, as they are sessile organisms that cannot escape from their environment, and they lack the effective thermoregulation mechanisms exhibited by homeotherms. Therefore, plants are expected to have developed adaptations to cope with heat stress [23]. To test our hypothesis, we grew *Arabidopsis thaliana* plants under normal (22 °C) and heat stress conditions (37 °C), and measured gene expression levels. Proteins overexpressed under heat stress were enriched in electrostatically charged amino acids and depleted in polar and hydrophobic amino acids. However, in contrast with our expectations, these proteins were also enriched in IDRs. These results indicate that *Arabidopsis* heat-induced proteins exploit some, but not all the same mechanisms as thermophile proteins to cope with high temperatures.

## 2. Results

### 2.1. Proteins That Are Overexpressed at High Temperatures Are Enriched in Electrostatically Charged Amino Acids and Depleted in Polar and Hydrophobic Amino Acids

**Figure 1:**
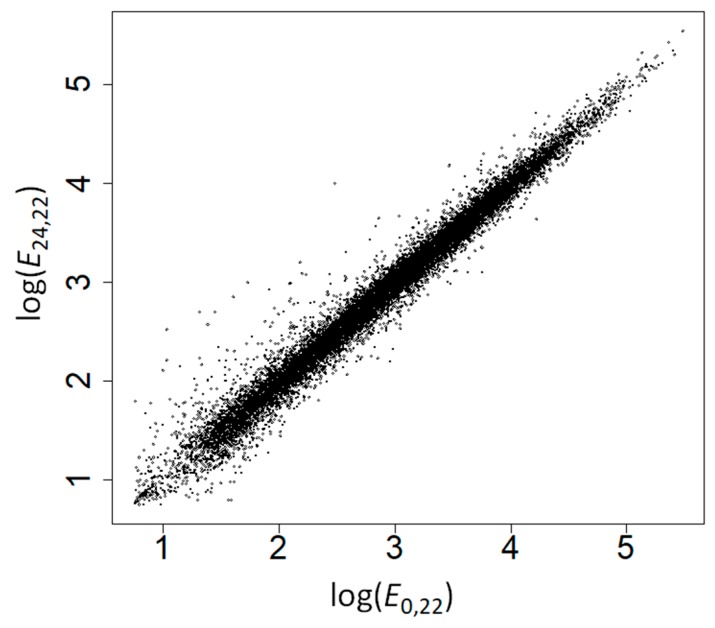
Correlation between gene expression levels at 22 °C at time 0 and at time 24 h.

**Figure 2:**
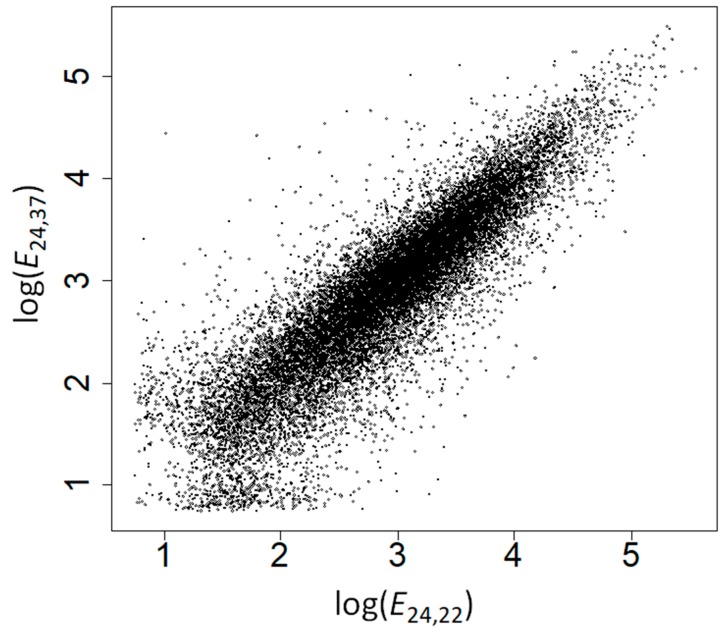
Correlation between gene expression levels at 22 °C at time 24 h and at 37 °C at time 24 h.

We grew *Arabidopsis* plants at 22 and 37 °C for 24 h, and performed microarray analyses to measure gene expression levels at the beginning of the experiment (*E*_0,22_ = expression at time 0 and 22 °C) and at the end of the experiment (*E*_24,22_ and *E*_24,37_). *E*_0,22_ strongly correlated with *E*_24,22_ (Spearman’s rank correlation coefficient, *ρ* = 0.991, *p* < 10^−200^; Figure 1) supporting the robustness of our gene expression measures—the small differences between gene expression at both time points could be due to differences in gene expression during development and to measurement errors. The correlation between *E*_24,22_ and *E*_24,37_ was weaker (*ρ* = 0.897, *p* = 10^−200^; Figure 2), highlighting the effect of heat stress on the expression of many genes.

For each gene with available probes (*n* = 20,491), we computed a response to heat stress (*R*) as the logarithm in base 2 of the ratio of expression levels at 37 and 22 °C (following formula 1). Genes with *R* > 0 are overexpressed at high temperatures, and genes with *R* < 0 are repressed. Genes with *R* > 1 (strongly overexpressed) are enriched in Gene Ontology biological processes “protein refolding”, “protein folding”, “chaperone cofactor-dependent protein refolding”, “chaperone-mediated protein folding”, “de novo posttranslational protein folding”, “de novo protein folding”, “cellular response to heat”, “response to heat”, “response to temperature stimulus”, and “heat acclimation”. They are also enriched in molecular functions “misfolded protein binding”, “heat shock protein binding”, “protein binding involved in protein folding”, and “unfolded protein binding” (Tables S1–S3).

**Figure 3:**
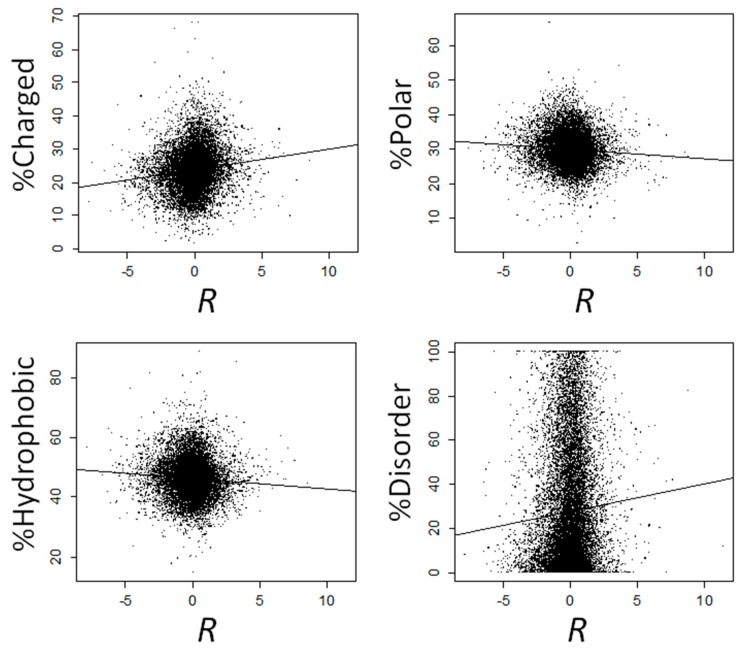
Correlations between response to high temperature (*R*) and the fraction of charged, polar, hydrophobic and disordered amino acids. Lines represent regression lines.

We observed a positive correlation between *R* and the fraction of charged amino acids (*ρ* = 0.146, *p* = 2.47 × 10^−98^), and negative correlations between *R* and both the fraction of polar (*ρ* = −0.076, *p* = 1.72 × 10^−27^) and hydrophobic (*ρ* = −0.084, *p* = 4.08 × 10^−33^) amino acids (Figure 3). We next computed the correlation between *R* and the frequency of each amino acid separately. The correlation was significantly positive for all four charged amino acids (Arg, Asp, Glu, and Lys), negative for all hydrophobic amino acids (significant for Gly, Ile, Phe, Pro, and Val), except Met (for which the correlation was non-significantly positive), and negative for all polar amino acids (significant for Asn, Ser, Thr, Trp and Tyr), except for Gln, for which the correlation was significantly positive (Table 1). All these correlations remained significant after controlling for multiple testing (Table 1).

Next, we compared the amino acid composition of proteins encoded by genes that are overexpressed (*R* > 0, *n* = 10,728) vs. proteins encoded by genes that are repressed (*R* < 0, *n* = 9763) at 37 °C. Overexpressed proteins were enriched in charged amino acids (median percent in overexpressed proteins: 24.32%; median percent in repressed proteins: 23.20%; Mann-Whitney’s *U* test, *p* = 1.90 × 10^−66^) and depleted in both polar (median percent in overexpressed proteins: 29.54%; median percent in repressed proteins: 30.04%; *p* = 2.53 × 10^−20^) and hydrophobic (median percent in overexpressed proteins: 45.77%; median percent in repressed proteins: 46.43%; *p* = 6.56 × 10^−21^) amino acids. In almost perfect agreement with our correlation analyses, proteins encoded by overexpressed genes were significantly enriched in Arg, Asp, Gln, Glu, and Lys, and significantly depleted in Asn, Gly, Ile, Phe, Pro, Ser, Thr, and Trp (Table 2).

Similar results were obtained when using a more stringent threshold to classify genes as overexpressed (*R* > 2, *n* = 826) or repressed (*R* < −2, *n* = 1214) at 37 °C. Overexpressed proteins are enriched in charged amino acids (median percent in overexpressed proteins: 25.30%; median percent in repressed proteins: 22.54%; *p* = 1.50 × 10^−26^) and depleted in both polar (median percent in overexpressed proteins: 29.74%; median percent in repressed proteins: 30.17%; *p* = 3.20 × 10^−8^) and hydrophobic (median percent in overexpressed proteins: 45.20%; median percent in repressed proteins: 47.24%; *p* = 6.04 × 10^−11^) amino acids. More specifically, overexpressed proteins are significantly enriched in Arg, Asp, Gln, Glu, and Lys, and significantly depleted in Asn, Cys, Gly, His, Ile, Phe, Pro, Thr, Trp, and Tyr (Table 3).

### 2.2. The Amino Acid Composition of Heat-Induced Proteins Is Not due to Covariation of Amino Acid Composition with GC Content, Gene Expression Levels, or Subcellular Location

We considered whether our results could be affected by confounding factors. First, GC content is known to affect amino acid composition [24], and *R* significantly correlates with GC content (*ρ* = 0.088, *p* = 9.76 × 10^−37^). Combined, these correlations alone might potentially explain the observed trends. To discard this possibility, we computed partial correlations between *R* and the frequency of each amino acid, while controlling for GC content, with very similar results. The correlation continued to be significantly positive for charged amino acids and significantly negative for polar and hydrophobic ones (Table 1). More specifically, the correlation was significantly positive for Arg, Asp, Gln, Glu, and Lys and significantly negative for Asn, Gly, Ile, Phe, Pro, Ser, Thr, Trp, Tyr, and Val. Both the negative correlation between *R* and Ala frequency and the positive correlation between *R* and Met frequency, which were initially not significant, became significant after controlling for GC content (Table 1).

Second, highly expressed proteins resemble proteins from thermophiles in their amino acid composition [25], and expression levels correlate with *R* (expression level at 22 °C: *ρ* = −0.156, *p* = 4.88 × 10^−112^; expression level at 37 °C: *ρ* = 0.241, *p* = 1.18 × 10^−268^). To discard the potential confounding effects of expression levels, we computed partial correlations between *R* and the frequency of each amino acid, while controlling for expression levels, again with very similar results. When controlling for expression levels at 22 °C, *R* correlated positively with the frequencies of Ala, Arg, Asp, Gln, Glu, and Lys and negatively with the frequencies of Asn, Cys, Gly, His, Ile, Leu, Phe, Pro, Ser, Thr, Trp, and Tyr. When controlling for expression levels at 37 °C, *R* correlated positively with the frequencies of Arg, Asp, Cys, Gln, Glu, Leu, Lys, and Met and negatively with the frequencies of Ala, Gly, Ile, Phe, Pro, Thr, Trp, Tyr, and Val. In both cases, the positive correlations between *R* and the frequency charged amino acids and the negative correlations between *R* and the frequencies of polar and hydrophobic amino acids remained significant (Table 1).

Proteins locating to different parts of the cell differ in their amino acid compositions and in their response to heat stress ([26,27]; Table 4). To discard subcellular location as a confounding factor, we analyzed the correlation between *R* and the amino acid composition separately for proteins locating to 10 different subcellular compartments (Table 5). The correlation between *R* and the fraction of charged amino acids was positive in nine of the compartments, which represents a significant departure from the 50% expected at random (one-tailed binomial test, *p* = 0.011). The correlation was significantly positive for the cytosol, the plastid (the compartments with the higher number of known/inferred proteins), and the mitochondrion. The correlation between *R* and the fraction of hydrophobic amino acids was negative in eight of the compartments (one-tailed binomial test, *p* = 0.055), significantly negative in the plastid and the mitochondrion, and significantly positive in the nucleus. The correlation between *R* and the fraction of polar amino acids was negative in half of the compartments, and significantly negative in the cytosol and the nucleus. These results suggest that the enrichment of heat-induced proteins in charged amino acids and their depletion in hydrophobic amino acids are not a byproduct of covariation of both *R* and amino acid composition with subcellular location. The lack of significance in most of the individual correlations is probably due to the low number of proteins for which location information is available, ranging from 720 for the plastid to 63 in the peroxisome (Table 4), which is expected to greatly reduce the statistical power of our compartment-specific analyses. However, we note an exception: among nuclear proteins *R* exhibits a significantly positive correlation with the percent of hydrophobic residues (Table 5).

### 2.3. Proteins That Are Overexpressed at High Temperatures Are Highly Disordered

For each *Arabidopsis* protein, we computed the percentage of amino acids that belong to IDRs using IUPred [28]. This percentage correlates positively with *R* (*ρ* = 0.059, *p* = 4.93 × 10^−17^; Figure 3). Genes that are overexpressed at 37 °C (*R* > 0) encode proteins that are more disordered than those that are repressed (*R* < 0), with median disorder percent of 19.19% and 16.51% for induced and repressed genes, respectively (Mann-Whitney’s *U* test, *p* = 2.01 × 10^−35^). The differences are more solid when comparing genes that are strongly overexpressed at 37 °C (*R* > 2) vs. those that are strongly repressed (*R* < −2), with percentages of median disorder of 21.54% and 11.51% for induced and repressed genes, respectively (Mann-Whitney’s *U* test, *P* = 2.03 × 10^−23^).

In agreement with previous works [29,30], we found a positive correlation between GC content and the percent of disordered residues (*ρ* = 0.044, *p* = 2.84 × 10^−10^). In addition, GC content positively correlates with *R* (*ρ* = 0.088, *p* = 9.76 × 10^−37^), making it possible that the positive correlation between *R* and disorder might be due to the covariation of both parameters with GC content. The correlation between *R* and disorder, however, is significant, even after controlling for GC content (*ρ* = 0.055, *p* = 3.44 × 10^−15^). 

Likewise, intrinsic disorder positively correlates with expression levels (at 22 °C: *ρ* = 0.040, *p* = 1.03 × 10^−8^; and at 37 °C: *ρ* = 0.072, *p* = 7.75 × 10^−25^), in agreement with previous results in *Escherichia coli* [31], but in contrast with observations in yeasts [32,33]. Disorder, however, significantly correlates with *R* after controlling for expression levels (at 22 °C: *ρ* = 0.066, *p* = 4.64 × 10^−21^; and at 37 °C: *ρ* = 0.043, *p* = 1.03 × 10^−9^).

Both intrinsic disorder and *R* substantially vary among proteins locating to different subcellular compartments (Table 4), thus raising the possibility that covariation of both factors with subcellular location may account for the observed enrichment of stress-induced proteins in IDRs. We analyzed the correlation between intrinsic disorder and *R* separately for proteins locating to 10 different subcellular compartments. The correlation was positive for eight of the tissues (significantly positive for the cytosol, endoplasmic reticulum, and the vacuole) and significantly negative for the nucleus and the plasma membrane (Table 5). These results indicate that the positive correlation between disorder and *R*, while generalized, does not apply to proteins locating to all compartments.

## 3. Discussion

We show that *Arabidopsis* proteins whose expression levels increase at high temperatures (heat-induced proteins) are enriched in charged amino acids, and depleted in polar and hydrophobic amino acids, compared to heat-repressed proteins. The enrichment of heat-induced proteins in charged amino acids and the depletion in polar amino acids are trends that mirror those observed in the proteins of thermophilic prokaryotes. The observed enrichment of heat-induced proteins in electrostatically charged amino acids was expected, as such amino acids can engage in salt bridges, which usually increase protein thermostability [1,2,3]—it should be noted, nonetheless, that not all charged amino acids participate in salt bridges, and that not all salt bridges increase thermostability [34]. However, the depletion of heat-induced proteins in hydrophobic amino acids was not expected, as the proteins of thermophilic prokaryotes are usually enriched in such amino acids (e.g., ref. [35]).

Despite the overall observed trends (heat-induced proteins being enriched in charged amino acids and depleted in polar and hydrophobic amino acids), not all amino acids vary according to these rules. In particular, the frequencies of Cys (polar), His (polar), Ala (hydrophobic), Leu (hydrophobic), and Met (hydrophobic) do not correlate significantly with *R*, and Gln (a polar amino acid) is more frequent in heat-induced proteins than in heat-repressed ones (Table 1). The enrichment of heat-induced proteins in Gln is surprising, given its tendency to undergo deamination at high temperatures [36].

We show that the observed overall trends are not due to heat-induced genes/proteins being different in terms of expression levels, GC content or subcellular location. When controlling for these factors, however, the direction of the correlations for certain amino acids change (Table 1). Thus, the observed trends in amino acid composition are likely the result of adaptation of heat-induced and heat-repressed *Arabidopsis* proteins to high and low temperatures, respectively.

Burra et al. [13] predicted that the proteins of thermophilic prokaryotes should be enriched in IDRs, as intrinsically disorder proteins are often resistant to high temperatures [16,17,18]. However, contradicting their predictions, they observed that thermophiles often are depleted in IDRs, which may compensate for the disorder induced by temperature. Similar observations were made in both another proteome-level analysis [15] and an analysis of FlgM proteins from bacteria adapted to different temperatures [14]. In agreement with Burra et al.’s prediction, we observed that *Arabidopsis* heat-induced proteins are enriched in IDRs. Our results suggest that there are different ways in which ordered/disordered regions can promote thermostability.

The correlations described in the current work are moderate, albeit statistically significant. Several scenarios may account for the weakness of the correlations. First, amino acid composition and protein intrinsic disorder may be affected by factors other than temperature. Second, the difference between the temperatures used in this study (22 vs. 37 °C) is small compared to the differences between the optimal temperatures of psychrophiles, mesophiles, and thermophiles. Third, certain plant genes may have changed their patterns of response to heat stress during the recent evolutionary history of *Arabidopsis*. i.e., certain genes that are currently heat-induced may have been heat-repressed in the past, and certain genes that are currently heat-repressed may have been heat-induced in the past. As amino acid and disorder adjustment to temperature is expected to take a relatively long amount of time, such switches in expression profiles may have limited the adaptation of proteomes to temperatures. Fourth, the adaptability of plant proteomes to temperatures may be more limited than that of prokaryotic proteomes, e.g., due to the higher complexity of protein-protein interaction networks and the smaller effective population size of plants [37].

In summary, the amino acid composition of heat-induced proteins in *Arabidopsis* mirrors to some extent, but not completely, that of the proteomes of thermophilic prokaryotes. This indicates that protein adaptation to high temperatures takes place partly through similar molecular mechanisms in prokaryotes and eukaryotes. Our observations also indicate that adaptation of proteins at the level of amino acid composition and protein intrinsic disorder can be detected not only when comparing the proteomes of species adapted to very different temperatures, but also among the proteins of the same species with different temperature response profiles. These observations expand our view of how eukaryotic proteomes adapt to different temperatures.

## 4. Materials and Methods

### 4.1. Plant Material, Growth Conditions, and Experimental Design

*Arabidopsis thaliana* Columbia ecotype seeds were sterilized with 70% ethanol for 20 min, 2.5% sodium hypochlorite (commercial bleach) with 0.05% Triton X-100 for 10 min, and finally, four washes with sterile dH_2_O. Seeds were placed onto Whatmann paper in Murashige and Skoog (MS) medium plates (Duchefa, Haarlem, The Netherlands). Plates were kept in the dark at 4 °C for 96 h for stratification, and incubated during 8 h in light at 22 °C to promote germination. Plates were transferred to darkness at 22 °C for 72 h. At this moment plates were either kept at 22 °C or transferred to 37 °C. Seedlings were harvested at 0 and 24 h with four biological replicates. Samples were frozen in liquid nitrogen and stored at −80 °C.

### 4.2. Microarray Analysis

Total RNA was extracted using the RNeasy Plant Mini Kit (Qiagen, Hilden, Germany), and RNA integrity was tested with the 2100 Bioanalyzer (Agilent, Santa Clara, CA, USA). Transcriptome analyses were carried out according to Minimum Information About a Microarray Experiment (MIAME) guidelines. We used the Agilent *Arabidopsis* (V4) Gene Expression 4 × 44K Microarray in a one-color experimental design. The microarray contained 43,803 probes (60-mer oligonucleotides). Four biological replicates were analyzed for each treatment (time points 0 and 24 h at 22 °C and 24 h at 37 °C).

Half a µg of RNA was amplified and labeled with the Agilent Low Input Quick Amp Labeling Kit. To assess the labeling and hybridization efficiencies we used an Agilent Spike-In Kit. Hybridization and slide washing were performed with the Gene Expression Hybridization Kit (Agilent) and Gene Expression Wash Buffers (Agilent), respectively. Then, slides were scanned at 5 µm resolution in an Agilent G2565AA microarray scanner, and image files were analyzed with the Feature Extraction software 9.5.1. We used the GeneSpring 12.1 software (Agilent) to perform the interarray analyses. To ensure a high-quality data set we removed control features, and selected only features for which the ‘IsWellAboveBG’ parameter was one in at least three out of four biological replicates (31,921 features from 43,803). Our microarray data sets have been submitted to the Gene Expression Omnibus database (accession number: GSE116592).

A new gene annotation of probes in the microarray was carried out using BLASTN searches (https://blast.ncbi.nlm.nih.gov/Blast.cgi), using the sequences of each probe as query against the Arabidopsis genome annotation in The Arabidopsis Information Resource (TAIR; www.arabidopsis.org), version 10. BLAST results for each probe were filtered with a minimum *E*-value of 9.9 × 10^−6^, a minimum sequence identity of 98% between probe and transcript, and a minimum overlap of the 75% of the probe sequence length. Probes matching multiple genes were not considered. Results for this gene annotation are quite similar to those obtained in similar analyses performed by TAIR (ftp://ftp.arabidopsis.org/Microarrays/Agilent/).

### 4.3. Gene Overexpression/Repression Analysis

For each probe and experimental condition (three conditions: 0 h at 22 °C, 24 h at 22 °C, and 24 h at 37 °C), expression levels were averaged across the four biological replicates. For those genes that mapped to more than one probe, expression levels were averaged across all probes. As a result, a single expression level was obtained for each gene and experimental condition.

For each gene with available probes (*n* = 20,491), the response (*R*) of its expression to heat stress was computed as:

(1) $$R=\log_{2}\frac{E_{24,\;37}}{E_{24,22}}$$

where *E*_24,37_ is expression level at 37 °C at 24 h, and *E*_24,22_ is expression level at 22 °C at 24 h. *R* takes positive values for genes that are overexpressed at 37 °C compared to 22 °C, and negative values for those that are repressed.

### 4.4. Protein and Gene Sequence Analysis

All *Arabidopsis* protein sequences were obtained from Ensembl Plants [38] (assembly: TAIR10). For each gene encoding multiple proteins (alternative splicing isoforms), the longest protein was selected for analysis. For each protein, the frequency of each amino acid was computed by dividing the number of occurrences of the amino acid by the length of the protein. GC content of each gene was retrieved from Ensembl Plants’ Biomart [38,39]. For each protein, the most likely subcellular location was retrieved from the SUBA4 database [40]. The consensus location was used. Only proteins located to a single compartment were used in compartment-specific analyses.

### 4.5. Prediction of Protein Intrinsic Disorder

Protein intrinsic disorder prediction was carried out using IUPred [28] for regions of disorder of at least 30 amino acids (“long” option). IUPred predicts tendency for polypeptide chains to be intrinsically disordered or ordered by analyzing the composition of amino acids within a window of 30 consecutive amino acids. It does so by utilizing an energy predictor matrix to estimate the tendency for pairs of amino acids to form strong stabilizing connections, the underlying assumption being that globular proteins form strong stabilizing contacts whereas structurally disordered proteins lack this capacity. IUPred reports a disorder score for each residue ranging from 0 to 1, conferring complete order to disorder, respectively. In this study, we used a threshold of >0.4 to calculate the proportion of amino acids within each protein that were likely to be in disordered regions.

### 4.6. Statistical Analyses

Statistical analyses were conducted using R [41]. Partial correlation analyses were conducted using the R function pcor.test [42]. Tests repeated on all 20 amino acids were corrected for multiple testing using the Benjamini-Hochberg approach [43].

## Figures and Tables

**Table 1:** Correlations between amino acid frequencies and response to high temperature.

Type	Amino Acid	No Control			Controlling for GC Content			Controlling for *E*_24,22_			Controlling for *E*_24,37_		
		*ρ*	*p*-Value	*q*-Value	*ρ*	*p*-Value	*q*-Value	*ρ*	*p*-Value	*q*-Value	*ρ*	*p*-Value	*q*-Value
Charged	Arg	0.075	**1.31 × 10^−26^**	**4.37 × 10^−26^**	0.068	**2.05 × 10^−22^**	**5.86 × 10^−22^**	0.068	**0.013**	**0.015**	0.079	**1.02 × 10^−29^**	**3.40 × 10^−29^**
	Asp	0.104	**1.62 × 10^−50^**	**1.62 × 10^−49^**	0.105	**9.95 × 10^−52^**	**9.95 × 10^−51^**	0.106	**7.16 × 10^−53^**	**7.16 × 10^−52^**	0.095	**1.84 × 10^−42^**	**1.23 × 10^−41^**
	Glu	0.118	**5.48 × 10^−64^**	**1.10 × 10^−62^**	0.122	**5.60 × 10^−69^**	**1.12 × 10^−67^**	0.115	**2.61 × 10^−61^**	**5.22 × 10^−60^**	0.115	**2.60 × 10^−61^**	**5.20 × 10^−60^**
	Lys	0.082	**8.23 × 10^−32^**	**4.12 × 10^−31^**	0.100	**9.58 × 10^−47^**	**4.79 × 10^−46^**	0.081	**2.27 × 10^−31^**	**9.08 × 10^−31^**	0.079	**8.76 × 10^−30^**	**3.40 × 10^−29^**
	Total	0.146	**2.47 × 10^−98^**		0.155	**3.61 × 10^−111^**		0.145	**1.04 × 10^−97^**		0.140	**1.65 × 10^−90^**	
Polar	Asn	−0.025	**3.86 × 10^−4^**	**0.001**	−0.018	**0.011**	**0.015**	−0.044	**4.17 × 10^−10^**	**8.34 × 10^−10^**	0.005	0.433	0.433
	Cys	−0.011	0.127	0.158	−0.009	0.187	0.208	−0.034	**1.54 × 10^−6^**	**2.57 × 10^−6^**	0.026	**2.07 × 10^−4^**	**3.19 × 10^−4^**
	Gln	0.046	**3.20 × 10^−11^**	**7.11 × 10^−11^**	0.053	**2.36 × 10^−14^**	**5.24 × 10^−14^**	0.046	**6.79 × 10^−11^**	**1.51 × 10^−10^**	0.044	**3.95 × 10^−10^**	**7.90 × 10^−10^**
	His	−0.010	0.134	0.158	−0.009	0.210	0.221	−0.024	**0.001**	**0.001**	0.010	0.146	0.154
	Ser	−0.036	**2.25 × 10^−7^**	**4.09 × 10^−7^**	−0.042	**2.36 × 10^−9^**	**4.72 × 10^−9^**	−0.052	**1.00 × 10^−13^**	**2.86 × 10^−13^**	−0.012	0.092	0.102
	Thr	−0.099	**1.10 × 10^−45^**	**7.33 × 10^−45^**	−0.100	**9.24 × 10^−47^**	**4.79 × 10^−46^**	−0.098	**1.12 × 10^−44^**	**7.47 × 10^−44^**	−0.096	**2.75 × 10^−43^**	**2.75 × 10^−42^**
	Trp	−0.033	**2.26 × 10^−6^**	**3.77 × 10^−6^**	−0.036	**2.50 × 10^−7^**	**4.55 × 10^−7^**	−0.039	**2.11 × 10^−8^**	**3.84 × 10^−8^**	−0.022	**0.002**	**0.002**
	Tyr	−0.024	**0.001**	**0.001**	−0.016	**0.021**	**0.026**	−0.025	**3.72 × 10^−4^**	**4.96 × 10^−4^**	−0.021	**0.003**	**0.004**
	Total	−0.076	**1.72 × 10^−27^**		−0.072	**1.11 × 10^−24^**		−0.102	**9.48 × 10^−49^**		−0.034	**9.25 × 10^−7^**	
Hydro phobic	Ala	−0.008	0.280	0.311	−0.020	**0.004**	**0.006**	0.027	**1.32 × 10^−4^**	**1.89 × 10^−4^**	−0.060	**1.50 × 10^−17^**	**3.75 × 10^−17^**
	Gly	−0.054	**1.40 × 10^−14^**	**3.50 × 10^−14^**	−0.066	**1.99 × 10^−21^**	**4.98 × 10^−21^**	−0.028	**5.46 × 10^−5^**	**8.40 × 10^−5^**	−0.092	**1.17 × 10^−39^**	**5.85 × 10^−39^**
	Ile	−0.045	**1.01 × 10^−10^**	**2.02 × 10^−10^**	−0.035	**5.63 × 10^−7^**	**9.38 × 10^−7^**	−0.052	**1.55 × 10^−13^**	**3.88 × 10^−13^**	−0.033	**2.91 × 10^−6^**	**5.29 × 10^−6^**
	Leu	−0.004	0.547	0.547	−0.004	0.533	0.533	−0.016	**0.021**	**0.023**	0.015	**0.029**	**0.034**
	Met	0.006	0.387	0.407	0.014	**0.042**	**0.049**	−0.001	0.942	0.942	0.017	**0.017**	**0.021**
	Phe	−0.075	**1.04 × 10^−26^**	**4.16 × 10^−26^**	−0.070	**9.79 × 10^−24^**	**3.26 × 10^−23^**	−0.084	**2.59 × 10^−33^**	**1.30 × 10^−32^**	−0.056	**1.36 × 10^−15^**	**3.02 × 10^−15^**
	Pro	−0.060	**8.03 × 10^−18^**	**2.29 × 10^−17^**	−0.074	**1.85 × 10^−26^**	**7.40 × 10^−26^**	−0.052	**8.41 × 10^−14^**	**2.80 × 10^−13^**	−0.070	**7.88 × 10^−24^**	**2.25 × 10^−23^**
	Val	−0.017	**0.012**	**0.017**	−0.024	**0.001**	**0.001**	−0.006	0.370	0.390	−0.033	**3.30 × 10^−6^**	**5.50 × 10^−6^**
	Total	−0.084	**4.08 × 10^−33^**		−0.096	**1.31 × 10^−43^**		−0.064	**2.88 × 10^−20^**		−0.109	**2.73 × 10^−55^**	

*p*-values and *q*-values shown in bold face represent significant tests at *α* = 0.05 or *q* = 0.05.

**Table 2:** Amino acid frequencies in overexpressed (*R* > 0) and repressed (*R* < 0) proteins at high temperatures.

Type	Amino Acid	Median Overexpressed (%)	Median Repressed (%)	*p*-Value	*q*-Value
Charged	Arg	5.43	5.19	**8.06 × 10^−21^**	**4.61 × 10^−20^**
	Asp	5.36	5.10	**1.60 × 10^−36^**	**6.40 × 10^−35^**
	Glu	6.61	6.15	**8.28 × 10^−44^**	**6.62 × 10^−42^**
	Lys	6.33	6.06	**1.20 × 10^−21^**	**8.00 × 10^−21^**
	Total	24.32	23.20	**1.90 × 10^−66^**	
Polar	Asn	4.08	4.12	**0.017**	**0.024**
	Cys	1.59	1.60	**0.043**	0.060
	Gln	3.27	3.16	**7.77 × 10^−8^**	**1.88 × 10^−7^**
	His	2.11	2.10	0.204	0.244
	Ser	8.79	8.96	**2.27 × 10^−7^**	**5.19 × 10^−7^**
	Thr	4.90	5.13	**5.31 × 10^−34^**	**1.42 × 10^−32^**
	Trp	1.07	1.11	**4.75 × 10^−4^**	**0.001**
	Tyr	2.65	2.68	0.132	0.163
	Total	29.54	30.04	**2.53 × 10^−20^**	
Hydrophobic	Ala	6.32	6.30	0.889	0.889
	Gly	6.18	6.41	**2.77 × 10^−10^**	**8.21 × 10^−10^**
	Ile	5.12	5.23	**1.87 × 10^−7^**	**4.40 × 10^−7^**
	Leu	9.24	9.27	0.675	0.720
	Met	2.38	2.37	0.399	0.449
	Phe	4.08	4.28	**1.55 × 10^−18^**	**7.75 × 10^−18^**
	Pro	4.54	4.71	**2.56 × 10^−12^**	**8.53 × 10^−12^**
	Val	6.67	6.68	0.178	0.215
	Total	45.77	46.43	**6.56 × 10^−21^**	

*p*-values correspond to the Mann-Whitney’s *U* test. *p*-values and *q*-values shown in bold face represent significant tests at α = 0.05 or *q* = 0.05.

**Table 3:** Amino acid frequencies in highly overexpressed (*R* > 2) and highly repressed (*R* < −2) proteins at high temperatures.

Type	Amino Acid	Median Overexpressed (%)	Median Repressed (%)	*p*-Value	*q*-Value
Charged	Arg	5.26	4.80	**7.82 × 10^−9^**	**1.04 × 10^−7^**
	Asp	5.51	4.95	**1.62 × 10^−12^**	**4.32 × 10^−11^**
	Glu	6.92	5.92	**1.31 × 10^−17^**	**1.05 × 10^−15^**
	Lys	6.78	6.17	**1.78 × 10^−7^**	**1.78 × 10^−6^**
	Total	25.30	22.54	**1.50 × 10^−26^**	
Polar	Asn	4.04	4.29	**2.81 × 10^−4^**	**0.001**
	Cys	1.66	1.69	**0.031**	**0.045**
	Gln	3.13	2.94	**2.87 × 10^−4^**	**6.57 × 10^−4^**
	His	2.03	2.12	**0.023**	**0.035**
	Ser	8.47	8.41	0.780	0.810
	Thr	4.95	5.26	**3.52 × 10^−6^**	**1.56 × 10^−5^**
	Trp	1.05	1.15	**0.035**	**0.050**
	Tyr	2.57	2.86	**6.09 × 10^−5^**	**1.87 × 10^−4^**
	Total	29.74	30.17	**3.20 × 10^−8^**	
Hydrophobic	Ala	6.11	6.12	0.867	0.878
	Gly	6.05	6.50	**5.49 × 10^−5^**	**1.76 × 10^−4^**
	Ile	5.25	5.48	**0.001**	**0.002**
	Leu	9.01	9.17	0.215	0.292
	Met	2.46	2.52	0.321	0.395
	Phe	4.12	4.65	**9.55 × 10^−13^**	**3.82 × 10^−11^**
	Pro	4.31	4.62	**9.34 × 10^−5^**	**2.58 × 10^−4^**
	Val	6.76	6.84	0.294	0.386
	Total	45.20	47.24	**6.04 × 10^−11^**	

*p*-values correspond to the Mann-Whitney’s *U* test. *p*-values and *q*-values shown in bold face represent significant tests at *α* = 0.05 or *q* = 0.05.

**Table 4:** Amino acid composition, intrinsic disorder and response to heat stress of proteins locating to different subcellular locations.

Subcellular Location	*n*	Median Charged Amino Acids (%)	Median Polar Amino Acids (%)	Median Hydrophobic Amino Acids (%)	Median Intrinsic Disorder (%)	Median *R*
Cytosol	633	25.46	26.74	47.31	15.64	0.131
Endoplasmic reticulum	163	24.12	27.22	48.68	10.11	0.147
Extracellular	197	18.94	32.87	48.43	8.61	−0.296
Golgi	375	23.20	29.37	47.38	14.22	0.108
Mitochondrion	286	23.12	27.63	49.26	14.93	0.261
Nucleus	446	26.50	29.16	43.92	42.73	0.406
Peroxisome	63	23.16	26.47	50.00	10.61	−0.207
Plasma membrane	343	22.21	28.63	48.73	14.97	−0.195
Plastid	720	23.33	27.65	48.91	15.28	−0.190
Vacuole	81	21.14	28.24	49.75	8.12	−0.008

**Table 5:** Correlations between amino acid frequencies and response to high temperature among proteins of different subcellular locations.

Subcellular Location	Correlation *R*-Charged Amino Acids		Correlation *R*-Polar Amino Acids		Correlation *R*-Hydrophobic Amino Acids		Correlation *R*-Intrinsic Disorder	
	*ρ*	*p*-Value	*ρ*	*p*-Value	*ρ*	*p*-Value	*ρ*	*p*-Value
Cytosol	0.171	**1.54 × 10^−5^**	−0.142	**3.27 × 10^−4^**	−0.069	0.082	0.123	**0.002**
Endoplasmic reticulum	0.061	0.437	−0.015	0.847	−0.112	0.155	0.226	**0.004**
Extracellular	0.054	0.452	0.021	0.765	−0.073	0.309	0.068	0.346
Golgi	−0.046	0.370	0.068	0.191	−0.009	0.866	0.073	0.156
Mitochondrion	0.124	**0.036**	0.060	0.312	−0.125	**0.034**	0.065	0.272
Nucleus	0.020	0.681	−0.119	**0.012**	0.102	**0.031**	−0.207	**1.09 × 10^−5^**
Peroxisome	0.016	0.902	−0.104	0.416	0.080	0.535	0.237	0.062
Plasma membrane	0.064	0.234	−0.017	0.750	−0.004	0.947	−0.154	**0.004**
Plastid	0.137	**2.20 × 10^−4^**	0.007	0.859	−0.110	**0.003**	0.062	0.095
Vacuole	0.184	0.099	0.082	0.466	−0.189	0.091	0.266	**0.017**

*p*-values shown in bold face represent significant tests at *α* = 0.05.

## Supplementary Materials

Supplementary materials can be found at http://www.mdpi.com/1422-0067/19/8/2276/s1.

## Abbreviations

IDR	Intrinsically Disordered Region
MS	Murashige and Skoog
TAIR	The Arabidopsis Information Resource
BLAST	Basic Local Alignment Search Tool
