# Supplementary material for: Arabidopsis Heat Stress-Induced Proteins Are Enriched in Electrostatically Charged Amino Acids and Intrinsically Disordered Regions
Source: Int J Mol Sci. 2018 Aug 3;19(8):2276. doi: 10.3390/ijms19082276 (PMC6121531; doi:10.3390/ijms19082276)
Supplement: Supplementary file 1 [file ijms-19-02276-s001.pdf]

Table S1. Gene Ontology biological process enrichment analysis of genes with *R* > 1.

GO category	# whole genome	# sample	Expected in sample	Fold enrichment	Sign enrichment	P-value	Q-value
polyadenylation-dependent snoRNA 3'-end processing	8	6	.92	6.54	+	1.70E-03	4.91E-02
snoRNA 3'-end processing	13	8	1.49	5.37	+	7.35E-04	2.48E-02
ncRNA 3'-end processing	32	18	3.67	4.91	+	1.11E-06	9.95E-05
ncRNA processing	343	139	39.31	3.54	+	2.41E-30	1.78E-27
ncRNA metabolic process	421	147	48.25	3.05	+	1.43E-26	7.71E-24
RNA metabolic process	3039	593	348.30	1.70	+	2.77E-33	3.28E-30
nucleic acid metabolic process	3438	682	394.03	1.73	+	8.10E-41	2.39E-37
nucleobase-containing compound metabolic process	3878	724	444.46	1.63	+	6.42E-36	9.49E-33
organic cyclic compound metabolic process	4493	770	514.94	1.50	+	7.00E-28	4.14E-25
organic substance metabolic process	9935	1270	1138.65	1.12	+	4.87E-06	3.60E-04
metabolic process	11121	1384	1274.58	1.09	+	1.90E-04	7.50E-03
cellular nitrogen compound metabolic process	4767	825	546.35	1.51	+	1.77E-31	1.50E-28
nitrogen compound metabolic process	8015	1122	918.60	1.22	+	1.47E-13	3.63E-11
cellular metabolic process	9524	1230	1091.54	1.13	+	1.24E-06	1.08E-04
cellular process	12555	1589	1438.93	1.10	+	4.25E-07	4.41E-05
heterocycle metabolic process	4196	753	480.90	1.57	+	1.20E-32	1.18E-29
cellular aromatic compound metabolic process	4370	761	500.85	1.52	+	2.59E-29	1.70E-26
primary metabolic process	9223	1201	1057.05	1.14	+	3.70E-07	4.05E-05
macromolecule metabolic process	7271	1052	833.33	1.26	+	4.19E-16	1.18E-13
RNA processing	819	269	93.87	2.87	+	5.49E-44	3.24E-40
gene expression	3289	556	376.95	1.47	+	2.75E-18	1.02E-15
RNA 3'-end processing	65	29	7.45	3.89	+	3.59E-08	5.45E-06
snoRNA processing	18	11	2.06	5.33	+	7.93E-05	3.61E-03
snoRNA metabolic process	20	12	2.29	5.24	+	4.30E-05	2.08E-03
protein refolding	26	17	2.98	5.70	+	4.68E-07	4.78E-05
protein folding	266	90	30.49	2.95	+	4.22E-16	1.13E-13
U4 snRNA 3'-end processing	11	7	1.26	5.55	+	1.38E-03	4.18E-02
snRNA 3'-end processing	15	11	1.72	6.40	+	2.37E-05	1.32E-03
snRNA processing	16	11	1.83	6.00	+	3.63E-05	1.82E-03
snRNA metabolic process	23	17	2.64	6.45	+	1.34E-07	1.72E-05
chiasma assembly	16	10	1.83	5.45	+	1.46E-04	5.93E-03
synapsis	28	14	3.21	4.36	+	4.66E-05	2.24E-03

chromosome organization involved in meiotic cell cycle	49	20	5.62	3.56	+	1.30E-05	7.79E-04
chromosome organization	544	113	62.35	1.81	+	5.38E-08	7.95E-06
organelle organization	1645	297	188.53	1.58	+	1.26E-12	2.86E-10
cellular component organization	2799	423	320.79	1.32	+	5.44E-08	7.84E-06
cellular component organization or biogenesis	3161	528	362.28	1.46	+	1.79E-16	5.28E-14
meiotic cell cycle process	145	36	16.62	2.17	+	1.08E-04	4.67E-03
meiotic cell cycle	173	43	19.83	2.17	+	2.32E-05	1.30E-03
reproductive process	1756	251	201.25	1.25	+	9.32E-04	3.05E-02
reproduction	1766	253	202.40	1.25	+	8.38E-04	2.77E-02
cell cycle	568	95	65.10	1.46	+	9.41E-04	3.06E-02
homologous chromosome segregation	35	15	4.01	3.74	+	9.98E-05	4.34E-03
meiotic chromosome segregation	58	19	6.65	2.86	+	2.31E-04	8.85E-03
meiotic nuclear division	100	29	11.46	2.53	+	5.29E-05	2.50E-03
nuclear division	156	40	17.88	2.24	+	2.53E-05	1.40E-03
organelle fission	201	48	23.04	2.08	+	2.07E-05	1.19E-03
nuclear chromosome segregation	100	29	11.46	2.53	+	5.29E-05	2.48E-03
chromosome segregation	124	36	14.21	2.53	+	5.47E-06	3.85E-04
meiosis I	72	24	8.25	2.91	+	2.94E-05	1.58E-03
meiosis I cell cycle process	77	26	8.82	2.95	+	1.16E-05	6.99E-04
reciprocal meiotic recombination	58	21	6.65	3.16	+	3.41E-05	1.75E-03
homologous recombination	58	21	6.65	3.16	+	3.41E-05	1.74E-03
DNA recombination	155	47	17.76	2.65	+	7.31E-08	9.83E-06
DNA metabolic process	537	111	61.55	1.80	+	6.76E-08	9.29E-06
cellular component assembly	788	146	90.31	1.62	+	3.11E-07	3.61E-05
cellular component biogenesis	1309	270	150.02	1.80	+	1.93E-17	6.02E-15
RNA phosphodiester bond hydrolysis, endonucleolytic	22	12	2.52	4.76	+	8.58E-05	3.84E-03
RNA phosphodiester bond hydrolysis	59	26	6.76	3.85	+	2.17E-07	2.68E-05
nucleic acid phosphodiester bond hydrolysis	86	33	9.86	3.35	+	7.65E-08	1.00E-05
spliceosomal snRNP assembly	28	15	3.21	4.67	+	1.35E-05	7.89E-04
mRNA splicing, via spliceosome	184	59	21.09	2.80	+	3.50E-10	6.67E-08
mRNA processing	393	109	45.04	2.42	+	3.54E-14	9.10E-12
mRNA metabolic process	523	143	59.94	2.39	+	9.97E-18	3.27E-15
RNA splicing, via transesterification reactions with bulged adenosine as nucleophile	201	67	23.04	2.91	+	4.16E-12	9.11E-10
RNA splicing, via transesterification reactions	201	67	23.04	2.91	+	4.16E-12	8.79E-10
RNA splicing	285	86	32.66	2.63	+	4.04E-13	9.55E-11
ribonucleoprotein complex assembly	212	54	24.30	2.22	+	1.19E-06	1.05E-04

ribonucleoprotein complex subunit organization	221	57	25.33	2.25	+	4.23E-07	4.47E-05
protein-containing complex subunit organization	645	132	73.92	1.79	+	7.06E-09	1.19E-06
ribonucleoprotein complex biogenesis	532	167	60.97	2.74	+	9.26E-26	4.56E-23
cellular protein-containing complex assembly	508	110	58.22	1.89	+	9.52E-09	1.56E-06
protein-containing complex assembly	554	115	63.49	1.81	+	3.47E-08	5.39E-06
endoplasmic reticulum unfolded protein response	19	10	2.18	4.59	+	4.14E-04	1.49E-02
signal transduction	1944	155	222.80	.70	-	2.63E-06	2.10E-04
signaling	1973	155	226.13	.69	-	9.92E-07	9.32E-05
cell communication	2207	174	252.94	.69	-	2.74E-07	3.24E-05
cellular response to unfolded protein	33	18	3.78	4.76	+	1.55E-06	1.27E-04
response to unfolded protein	36	20	4.13	4.85	+	3.30E-07	3.76E-05
response to topologically incorrect protein	55	22	6.30	3.49	+	6.35E-06	4.27E-04
cellular response to topologically incorrect protein	48	20	5.50	3.64	+	1.02E-05	6.25E-04
cellular response to stress	967	162	110.83	1.46	+	1.31E-05	7.75E-04
maturation of LSU-rRNA	40	21	4.58	4.58	+	3.45E-07	3.85E-05
ribosomal large subunit biogenesis	104	37	11.92	3.10	+	6.25E-08	8.80E-06
ribosome biogenesis	440	138	50.43	2.74	+	1.89E-21	7.46E-19
rRNA processing	228	99	26.13	3.79	+	1.03E-23	4.37E-21
rRNA metabolic process	241	102	27.62	3.69	+	1.01E-23	4.58E-21
RNA secondary structure unwinding	27	14	3.09	4.52	+	3.43E-05	1.73E-03
Group II intron splicing	18	9	2.06	4.36	+	1.06E-03	3.39E-02
cleavage involved in rRNA processing	24	12	2.75	4.36	+	1.61E-04	6.48E-03
rRNA catabolic process	19	9	2.18	4.13	+	1.42E-03	4.29E-02
ncRNA catabolic process	22	10	2.52	3.97	+	9.99E-04	3.23E-02
RNA catabolic process	119	29	13.64	2.13	+	5.96E-04	2.09E-02
RNA surveillance	19	9	2.18	4.13	+	1.42E-03	4.27E-02
mitochondrial mRNA modification	45	20	5.16	3.88	+	4.79E-06	3.59E-04
mRNA modification	62	25	7.11	3.52	+	1.36E-06	1.13E-04
RNA modification	441	176	50.54	3.48	+	1.75E-37	3.46E-34
macromolecule modification	2842	406	325.72	1.25	+	1.85E-05	1.07E-03
mitochondrial RNA modification	48	22	5.50	4.00	+	1.09E-06	1.01E-04
mitochondrial RNA metabolic process	65	31	7.45	4.16	+	3.61E-09	6.27E-07
chaperone cofactor-dependent protein refolding	34	15	3.90	3.85	+	7.71E-05	3.59E-03
chaperone-mediated protein folding	60	25	6.88	3.64	+	8.41E-07	8.16E-05
'de novo' posttranslational protein folding	35	16	4.01	3.99	+	3.20E-05	1.69E-03
'de novo' protein folding	46	23	5.27	4.36	+	1.90E-07	2.39E-05
maturation of 5.8S rRNA	32	14	3.67	3.82	+	1.42E-04	5.80E-03
rRNA modification	40	17	4.58	3.71	+	3.81E-05	1.89E-03
protein import into mitochondrial matrix	29	12	3.32	3.61	+	6.24E-04	2.16E-02

protein targeting to mitochondrion	52	21	5.96	3.52	+	9.11E-06	5.79E-04
establishment of protein localization to mitochondrion	52	21	5.96	3.52	+	9.11E-06	5.67E-04
protein localization to mitochondrion	52	21	5.96	3.52	+	9.11E-06	5.73E-04
mitochondrial transport	128	30	14.67	2.04	+	8.85E-04	2.91E-02
mitochondrion organization	171	60	19.60	3.06	+	9.40E-12	1.92E-09
intracellular protein transmembrane transport	85	23	9.74	2.36	+	6.56E-04	2.23E-02
protein transmembrane transport	88	23	10.09	2.28	+	1.36E-03	4.16E-02
protein transmembrane import into intracellular organelle	53	17	6.07	2.80	+	5.88E-04	2.07E-02
protein import	133	33	15.24	2.16	+	2.57E-04	9.79E-03
mitochondrial transmembrane transport	56	17	6.42	2.65	+	1.34E-03	4.12E-02
maturation of SSU-rRNA from tricistronic rRNA transcript (SSU-rRNA, 5.8S rRNA, LSU-rRNA)	44	18	5.04	3.57	+	3.40E-05	1.76E-03
maturation of SSU-rRNA	59	24	6.76	3.55	+	1.94E-06	1.57E-04
ribosomal small subunit biogenesis	95	32	10.89	2.94	+	1.25E-06	1.07E-04
nuclear-transcribed mRNA catabolic process, exonucleolytic	30	12	3.44	3.49	+	7.93E-04	2.65E-02
nuclear-transcribed mRNA catabolic process	90	23	10.31	2.23	+	1.51E-03	4.47E-02
cellular response to heat	65	26	7.45	3.49	+	9.49E-07	9.05E-05
response to heat	213	83	24.41	3.40	+	7.73E-18	2.69E-15
response to temperature stimulus	601	133	68.88	1.93	+	9.78E-11	1.93E-08
heat acclimation	43	17	4.93	3.45	+	7.83E-05	3.59E-03
mitochondrial gene expression	41	16	4.70	3.40	+	1.42E-04	5.84E-03
RNA methylation	64	22	7.34	3.00	+	4.26E-05	2.08E-03
macromolecule methylation	167	42	19.14	2.19	+	2.77E-05	1.52E-03
megagametogenesis	67	23	7.68	3.00	+	2.93E-05	1.59E-03
embryo sac development	136	45	15.59	2.89	+	1.79E-08	2.86E-06
gametophyte development	423	83	48.48	1.71	+	2.25E-05	1.28E-03
regulation of mRNA splicing, via spliceosome	38	13	4.36	2.98	+	1.57E-03	4.61E-02
regulation of RNA splicing	43	15	4.93	3.04	+	5.98E-04	2.08E-02
tRNA modification	72	24	8.25	2.91	+	2.94E-05	1.57E-03
tRNA processing	104	34	11.92	2.85	+	1.34E-06	1.13E-04
tRNA metabolic process	176	41	20.17	2.03	+	1.15E-04	4.94E-03
ribonucleoprotein complex export from nucleus	59	19	6.76	2.81	+	2.76E-04	1.04E-02
protein export from nucleus	64	19	7.34	2.59	+	7.58E-04	2.55E-02
nuclear export	75	26	8.60	3.02	+	7.92E-06	5.20E-04
nucleocytoplasmic transport	125	33	14.33	2.30	+	9.93E-05	4.35E-03
nuclear transport	125	33	14.33	2.30	+	9.93E-05	4.38E-03
RNA export from nucleus	70	26	8.02	3.24	+	2.87E-06	2.24E-04
RNA transport	97	31	11.12	2.79	+	5.36E-06	3.86E-04

establishment of RNA localization	97	31	11.12	2.79	+	5.36E-06	3.91E-04
RNA localization	104	33	11.92	2.77	+	2.87E-06	2.26E-04
nucleic acid transport	97	31	11.12	2.79	+	5.36E-06	3.82E-04
nucleobase-containing compound transport	147	36	16.85	2.14	+	1.26E-04	5.27E-03
protein-containing complex localization	72	20	8.25	2.42	+	1.46E-03	4.36E-02
ribonucleoprotein complex localization	59	19	6.76	2.81	+	2.76E-04	1.03E-02
response to high light intensity	80	24	9.17	2.62	+	1.28E-04	5.34E-03
response to hydrogen peroxide	71	21	8.14	2.58	+	3.99E-04	1.45E-02
mRNA transport	64	18	7.34	2.45	+	1.60E-03	4.65E-02
double-strand break repair via homologous recombination	76	21	8.71	2.41	+	1.07E-03	3.39E-02
recombinational repair	82	23	9.40	2.45	+	5.04E-04	1.80E-02
DNA repair	322	61	36.90	1.65	+	6.51E-04	2.23E-02
cellular response to DNA damage stimulus	351	68	40.23	1.69	+	1.75E-04	6.95E-03
double-strand break repair	113	31	12.95	2.39	+	7.79E-05	3.60E-03
protein-DNA complex subunit organization	98	24	11.23	2.14	+	1.57E-03	4.59E-02
embryo development ending in seed dormancy	557	109	63.84	1.71	+	1.11E-06	1.01E-04
embryo development	574	112	65.79	1.70	+	8.32E-07	8.20E-05
seed development	717	136	82.18	1.65	+	2.26E-07	2.73E-05
fruit development	753	140	86.30	1.62	+	4.19E-07	4.50E-05
post-embryonic development	1486	216	170.31	1.27	+	1.01E-03	3.25E-02
Unclassified	4505	421	516.32	.82	-	8.30E-06	5.33E-04
cellular response to hormone stimulus	966	77	110.71	.70	-	1.28E-03	4.02E-02
cellular response to endogenous stimulus	974	78	111.63	.70	-	1.35E-03	4.12E-02
response to endogenous stimulus	1823	163	208.93	.78	-	1.33E-03	4.10E-02
response to hormone	1815	162	208.02	.78	-	1.29E-03	4.04E-02
lipid metabolic process	1033	80	118.39	.68	-	3.50E-04	1.28E-02
carboxylic acid metabolic process	999	74	114.50	.65	-	1.19E-04	5.08E-03
oxoacid metabolic process	1156	86	132.49	.65	-	3.91E-05	1.93E-03
organic acid metabolic process	1159	86	132.83	.65	-	3.25E-05	1.70E-03
small molecule metabolic process	1751	146	200.68	.73	-	7.97E-05	3.60E-03
cofactor metabolic process	600	43	68.77	.63	-	1.61E-03	4.67E-02
small molecule biosynthetic process	757	53	86.76	.61	-	2.13E-04	8.28E-03
intracellular signal transduction	642	41	73.58	.56	-	9.50E-05	4.22E-03
vesicle-mediated transport	499	30	57.19	.52	-	2.15E-04	8.30E-03
secondary metabolic process	521	31	59.71	.52	-	1.24E-04	5.24E-03
carbohydrate biosynthetic process	337	19	38.62	.49	-	1.11E-03	3.51E-02
carbohydrate metabolic process	1067	73	122.29	.60	-	4.46E-06	3.38E-04
defense response to fungus	488	27	55.93	.48	-	5.19E-05	2.48E-03
response to fungus	571	31	65.44	.47	-	6.87E-06	4.57E-04

response to other organism	1209	96	138.56	.69	-	2.72E-04	1.03E-02
response to external biotic stimulus	1212	97	138.91	.70	-	3.33E-04	1.22E-02
response to external stimulus	1611	136	184.64	.74	-	3.00E-04	1.11E-02
response to biotic stimulus	1250	99	143.26	.69	-	1.67E-04	6.67E-03
multi-organism process	1728	153	198.05	.77	-	1.30E-03	4.05E-02
defense response to other organism	903	60	103.49	.58	-	9.23E-06	5.68E-04
defense response	1583	131	181.43	.72	-	1.39E-04	5.73E-03
plant-type cell wall organization or biogenesis	284	15	32.55	.46	-	1.49E-03	4.44E-02
cell wall organization or biogenesis	735	53	84.24	.63	-	5.51E-04	1.96E-02
protein phosphorylation	953	50	109.22	.46	-	1.29E-09	2.31E-07
phosphorylation	1283	77	147.04	.52	-	7.84E-10	1.45E-07
phosphate-containing compound metabolic process	1844	146	211.34	.69	-	3.71E-06	2.85E-04
phosphorus metabolic process	1917	148	219.71	.67	-	5.70E-07	5.71E-05
killing of cells of other organism	280	9	32.09	.28	-	6.02E-06	4.09E-04
cell killing	280	9	32.09	.28	-	6.02E-06	4.19E-04
disruption of cells of other organism	280	9	32.09	.28	-	6.02E-06	4.14E-04
modification of morphology or physiology of other organism	311	16	35.64	.45	-	6.49E-04	2.23E-02
interspecies interaction between organisms	372	21	42.63	.49	-	5.81E-04	2.06E-02
pectin metabolic process	175	5	20.06	.25	-	2.86E-04	1.06E-02
galacturonan metabolic process	176	5	20.17	.25	-	1.95E-04	7.63E-03
polysaccharide metabolic process	474	30	54.33	.55	-	8.28E-04	2.75E-02

Gene Ontology enrichment analyses were conducted using the “Enrichment analysis” tool in the Gene Ontology website (http://geneontology.org/). Indentation represents the hierarchy of GO terms.

Table S2. Gene Ontology molecular function enrichment analysis of genes with *R* > 1.

GO category	# whole genome	# sample	Expected in sample	Fold enrichment	Sign enrichment	P-value	Q-value
snoRNA binding	27	18	3.09	5.82	+	1.75E-07	3.07E-05
RNA binding	1535	397	175.93	2.26	+	1.15E-43	3.62E-40
nucleic acid binding	4092	722	468.98	1.54	+	4.19E-29	6.63E-26
organic cyclic compound binding	7074	996	810.75	1.23	+	3.19E-12	1.26E-09
binding	11441	1470	1311.25	1.12	+	6.95E-08	1.29E-05
heterocyclic compound binding	7055	995	808.57	1.23	+	2.25E-12	1.02E-09
RNA polymerase I activity	24	14	2.75	5.09	+	1.27E-05	1.29E-03
DNA-directed 5'-3' RNA polymerase activity	81	27	9.28	2.91	+	9.62E-06	1.05E-03
5'-3' RNA polymerase activity	89	29	10.20	2.84	+	9.26E-06	1.08E-03
RNA polymerase activity	89	29	10.20	2.84	+	9.26E-06	1.04E-03
catalytic activity, acting on RNA	435	116	49.86	2.33	+	5.69E-14	4.49E-11
nucleotidyltransferase activity	222	54	25.44	2.12	+	3.41E-06	4.15E-04
ATPase regulator activity	16	9	1.83	4.91	+	5.59E-04	3.27E-02
misfolded protein binding	16	9	1.83	4.91	+	5.59E-04	3.21E-02
heat shock protein binding	36	20	4.13	4.85	+	3.30E-07	4.75E-05
pre-mRNA binding	20	11	2.29	4.80	+	1.60E-04	1.20E-02
RNA polymerase III activity	31	16	3.55	4.50	+	1.00E-05	1.05E-03
protein binding involved in protein folding	33	16	3.78	4.23	+	1.82E-05	1.60E-03
unfolded protein binding	104	49	11.92	4.11	+	1.70E-13	1.07E-10
3'-5'-exoribonuclease activity	36	14	4.13	3.39	+	3.75E-04	2.32E-02
3'-5' exonuclease activity	73	22	8.37	2.63	+	2.41E-04	1.77E-02
exonuclease activity	113	31	12.95	2.39	+	7.79E-05	6.31E-03
nuclease activity	378	109	43.32	2.52	+	3.85E-15	4.05E-12
hydrolase activity, acting on ester bonds	1224	184	140.28	1.31	+	6.30E-04	3.49E-02
hydrolase activity	3503	480	401.48	1.20	+	1.13E-04	8.89E-03
exoribonuclease activity, producing 5'-phosphomonoesters	40	15	4.58	3.27	+	3.22E-04	2.07E-02
exoribonuclease activity	40	15	4.58	3.27	+	3.22E-04	2.03E-02
ribonuclease activity	108	28	12.38	2.26	+	3.18E-04	2.09E-02
RNA methyltransferase activity	60	20	6.88	2.91	+	1.32E-04	1.01E-02
endonuclease activity	260	81	29.80	2.72	+	5.06E-13	2.66E-10
ATP-dependent RNA helicase activity	66	20	7.56	2.64	+	4.60E-04	2.80E-02
ATP-dependent helicase activity	134	42	15.36	2.73	+	2.56E-07	4.05E-05
purine NTP-dependent helicase activity	134	42	15.36	2.73	+	2.56E-07	3.86E-05
helicase activity	203	61	23.27	2.62	+	1.22E-09	3.86E-07
nucleoside-triphosphatase activity	912	171	104.52	1.64	+	1.29E-08	3.14E-06
pyrophosphatase activity	962	175	110.25	1.59	+	5.03E-08	1.13E-05

hydrolase activity, acting on acid anhydrides, in phosphorus-containing anhydrides	974	176	111.63	1.58	+	6.15E-08	1.30E-05
hydrolase activity, acting on acid anhydrides	980	177	112.32	1.58	+	6.52E-08	1.29E-05
ATPase activity, coupled	499	94	57.19	1.64	+	2.11E-05	1.76E-03
ATPase activity	649	125	74.38	1.68	+	4.01E-07	5.50E-05
RNA-dependent ATPase activity	67	21	7.68	2.73	+	2.54E-04	1.78E-02
RNA helicase activity	67	21	7.68	2.73	+	2.54E-04	1.82E-02
Unclassified	4621	435	529.61	.82	-	1.30E-05	1.24E-03
cofactor binding	1000	78	114.61	.68	-	6.08E-04	3.43E-02
solute:proton symporter activity	163	5	18.68	.27	-	7.70E-04	4.20E-02
solute:cation symporter activity	176	6	20.17	.30	-	8.11E-04	4.34E-02
symporter activity	210	8	24.07	.33	-	4.61E-04	2.75E-02
secondary active transmembrane transporter activity	399	22	45.73	.48	-	2.58E-04	1.77E-02
transmembrane receptor protein serine/threonine kinase activity	171	3	19.60	.15	-	1.50E-05	1.39E-03
protein serine/threonine kinase activity	946	57	108.42	.53	-	1.95E-07	3.24E-05
protein kinase activity	1091	65	125.04	.52	-	1.19E-08	3.13E-06
kinase activity	1466	93	168.02	.55	-	1.02E-09	3.59E-07
catalytic activity, acting on a protein	2728	250	312.66	.80	-	2.92E-04	1.96E-02
phosphotransferase activity, alcohol group as acceptor	1246	76	142.80	.53	-	3.45E-09	9.92E-07
transmembrane receptor protein kinase activity	186	4	21.32	.19	-	2.03E-05	1.73E-03
transmembrane signaling receptor activity	225	6	25.79	.23	-	1.60E-05	1.45E-03
signaling receptor activity	254	6	29.11	.21	-	9.89E-07	1.30E-04
molecular transducer activity	295	9	33.81	.27	-	2.35E-06	2.97E-04

Gene Ontology enrichment analyses were conducted using the “Enrichment analysis” tool in the Gene Ontology website (http://geneontology.org/). Indentation represents the hierarchy of GO terms.

Table S3. Gene Ontology subcellular location enrichment analysis of genes with *R* > 1.

GO category	# whole genome	# sample	Expected in sample	Fold enrichment	Sign enrichment	P-value	Q-value
exon-exon junction complex	6	5	.69	7.27	+	3.11E-03	3.36E-02
nuclear part	1448	371	165.96	2.24	+	6.00E-40	6.43E-37
nucleus	9858	1308	1129.82	1.16	+	5.71E-10	1.80E-08
intracellular membrane-bounded organelle	17700	2342	2028.59	1.15	+	8.42E-30	9.01E-28
intracellular organelle	18054	2380	2069.17	1.15	+	6.31E-30	7.51E-28
organelle	18092	2380	2073.52	1.15	+	3.50E-29	3.40E-27
intracellular part	20055	2600	2298.50	1.13	+	2.95E-33	5.27E-31
intracellular	20080	2605	2301.37	1.13	+	8.47E-34	1.82E-31
cell part	22071	2746	2529.55	1.09	+	3.58E-22	2.25E-20
cell	22072	2746	2529.67	1.09	+	3.58E-22	2.13E-20
membrane-bounded organelle	17800	2344	2040.06	1.15	+	2.91E-28	2.39E-26
intracellular organelle part	5484	814	628.52	1.30	+	4.23E-14	1.97E-12
organelle part	5491	815	629.32	1.30	+	4.32E-14	1.93E-12
protein-containing complex	3227	581	369.85	1.57	+	1.06E-24	8.11E-23
90S preribosome	27	22	3.09	7.11	+	5.38E-10	1.75E-08
preribosome	87	53	9.97	5.32	+	4.90E-18	2.63E-16
ribonucleoprotein complex	829	196	95.01	2.06	+	5.63E-18	2.87E-16
preribosome, large subunit precursor	20	13	2.29	5.67	+	1.10E-05	2.11E-04
box C/D snoRNP complex	11	7	1.26	5.55	+	1.38E-03	1.54E-02
small nucleolar ribonucleoprotein complex	43	23	4.93	4.67	+	7.46E-08	2.00E-06
nucleolar part	73	45	8.37	5.38	+	1.13E-15	5.48E-14
nucleolus	477	172	54.67	3.15	+	2.67E-32	4.08E-30
nuclear lumen	1105	287	126.64	2.27	+	1.28E-31	1.72E-29
intracellular organelle lumen	1335	348	153.00	2.27	+	1.01E-38	5.39E-36
organelle lumen	1335	348	153.00	2.27	+	1.01E-38	2.70E-36
membrane-enclosed lumen	1335	348	153.00	2.27	+	1.01E-38	3.60E-36
intracellular non-membrane-bounded organelle	1721	354	197.24	1.79	+	1.34E-22	9.58E-21
non-membrane-bounded organelle	1721	354	197.24	1.79	+	1.34E-22	8.98E-21
small-subunit processome	51	32	5.85	5.47	+	1.03E-11	4.23E-10
cytoplasmic exosome (RNase complex)	15	9	1.72	5.24	+	3.93E-04	4.79E-03
exosome (RNase complex)	19	11	2.18	5.05	+	1.14E-04	1.71E-03
exoribonuclease complex	19	11	2.18	5.05	+	1.14E-04	1.69E-03
catalytic complex	1185	221	135.81	1.63	+	1.03E-10	3.54E-09
cytoplasmic part	10807	1432	1238.59	1.16	+	3.83E-11	1.46E-09

cytoplasm	13346	1752	1529.58	1.15	+	6.68E-14	2.86E-12
nuclear exosome (RNase complex)	15	9	1.72	5.24	+	3.93E-04	4.74E-03
DNA-directed RNA polymerase I complex	24	14	2.75	5.09	+	1.27E-05	2.38E-04
nuclear DNA-directed RNA polymerase complex	135	35	15.47	2.26	+	6.25E-05	9.99E-04
DNA-directed RNA polymerase complex	145	39	16.62	2.35	+	1.29E-05	2.39E-04
RNA polymerase complex	151	41	17.31	2.37	+	6.29E-06	1.30E-04
transferase complex, transferring phosphorus-containing groups	235	54	26.93	2.00	+	1.47E-05	2.62E-04
transferase complex	673	133	77.13	1.72	+	3.75E-08	1.06E-06
U5 snRNP	21	12	2.41	4.99	+	6.12E-05	1.02E-03
spliceosomal snRNP complex	82	30	9.40	3.19	+	6.63E-07	1.65E-05
small nuclear ribonucleoprotein complex	82	30	9.40	3.19	+	6.63E-07	1.61E-05
Sm-like protein family complex	85	30	9.74	3.08	+	1.22E-06	2.90E-05
DNA-directed RNA polymerase III complex	32	16	3.67	4.36	+	1.36E-05	2.46E-04
nucleoplasm part	414	85	47.45	1.79	+	3.38E-06	7.11E-05
nucleoplasm	518	110	59.37	1.85	+	2.65E-08	7.88E-07
anaphase-promoting complex	14	7	1.60	4.36	+	3.82E-03	3.93E-02
cullin-RING ubiquitin ligase complex	229	49	26.25	1.87	+	2.07E-04	2.84E-03
ubiquitin ligase complex	301	59	34.50	1.71	+	3.17E-04	3.99E-03
U1 snRNP	27	12	3.09	3.88	+	3.76E-04	4.68E-03
U4/U6 x U5 tri-snRNP complex	32	13	3.67	3.54	+	4.31E-04	5.13E-03
spliceosomal tri-snRNP complex	42	17	4.81	3.53	+	6.20E-05	1.02E-03
endoplasmic reticulum lumen	38	15	4.36	3.44	+	2.06E-04	2.86E-03
mitochondrial inner membrane presequence translocase complex	33	13	3.78	3.44	+	5.45E-04	6.35E-03
inner mitochondrial membrane protein complex	176	43	20.17	2.13	+	2.91E-05	5.03E-04
mitochondrial inner membrane	316	63	36.22	1.74	+	1.60E-04	2.29E-03
mitochondrial membrane	393	82	45.04	1.82	+	2.82E-06	6.04E-05
membrane	8477	792	971.55	.82	-	2.37E-11	9.41E-10
mitochondrial envelope	408	85	46.76	1.82	+	2.02E-06	4.51E-05
mitochondrial part	562	123	64.41	1.91	+	7.03E-10	2.15E-08
mitochondrion	3426	629	392.65	1.60	+	5.89E-29	5.26E-27
organelle envelope	1219	177	139.71	1.27	+	3.34E-03	3.54E-02
envelope	1219	177	139.71	1.27	+	3.34E-03	3.57E-02
organelle inner membrane	401	68	45.96	1.48	+	3.54E-03	3.71E-02
mitochondrial membrane part	229	50	26.25	1.91	+	9.59E-05	1.51E-03
membrane part	5721	531	655.68	.81	-	1.15E-07	2.92E-06
mitochondrial protein complex	255	65	29.23	2.22	+	8.17E-08	2.13E-06

catalytic step 2 spliceosome	61	23	6.99	3.29	+	8.64E-06	1.75E-04
spliceosomal complex	159	56	18.22	3.07	+	4.05E-11	1.50E-09
condensed nuclear chromosome	39	14	4.47	3.13	+	7.16E-04	8.16E-03
condensed chromosome	66	22	7.56	2.91	+	6.21E-05	1.01E-03
U2 snRNP	37	13	4.24	3.07	+	1.29E-03	1.45E-02
U2-type spliceosomal complex	57	19	6.53	2.91	+	1.92E-04	2.70E-03
lipid droplet	33	11	3.78	2.91	+	4.13E-03	4.21E-02
nuclear speck	84	25	9.63	2.60	+	1.01E-04	1.57E-03
nuclear body	113	35	12.95	2.70	+	2.11E-06	4.61E-05
nuclear pore	75	22	8.60	2.56	+	3.11E-04	3.97E-03
nuclear envelope	142	30	16.27	1.84	+	3.57E-03	3.72E-02
Cul4-RING E3 ubiquitin ligase complex	117	31	13.41	2.31	+	1.09E-04	1.66E-03
mitochondrial matrix	162	38	18.57	2.05	+	2.36E-04	3.16E-03
Unclassified	2402	198	275.29	.72	-	1.35E-06	3.13E-05
trans-Golgi network	314	16	35.99	.44	-	4.96E-04	5.84E-03
Golgi subcompartment	603	34	69.11	.49	-	9.01E-06	1.79E-04
Golgi apparatus part	627	37	71.86	.51	-	1.83E-05	3.21E-04
Golgi apparatus	1228	90	140.74	.64	-	1.02E-05	1.99E-04
organelle subcompartment	1529	125	175.24	.71	-	1.25E-04	1.84E-03
Golgi membrane	405	20	46.42	.43	-	3.72E-05	6.33E-04
extracellular region part	191	9	21.89	.41	-	4.73E-03	4.74E-02
extracellular region	2927	187	335.46	.56	-	1.16E-18	6.54E-17
integral component of plasma membrane	260	11	29.80	.37	-	2.62E-04	3.42E-03
integral component of membrane	4973	450	569.95	.79	-	6.58E-08	1.81E-06
intrinsic component of membrane	5222	473	598.49	.79	-	2.98E-08	8.64E-07
intrinsic component of plasma membrane	439	27	50.31	.54	-	6.98E-04	8.03E-03
plasma membrane part	590	44	67.62	.65	-	4.39E-03	4.43E-02
plasma membrane	3912	318	448.35	.71	-	4.59E-11	1.64E-09
cell periphery	4562	390	522.85	.75	-	4.27E-10	1.43E-08
endosome membrane	169	5	19.37	.26	-	3.82E-04	4.71E-03
endosomal part	173	5	19.83	.25	-	2.74E-04	3.54E-03
endosome	437	24	50.08	.48	-	1.38E-04	2.00E-03
cytoplasmic vesicle	607	44	69.57	.63	-	2.11E-03	2.33E-02
intracellular vesicle	610	45	69.91	.64	-	2.73E-03	2.98E-02
vesicle	667	45	76.44	.59	-	2.22E-04	3.01E-03
cytoplasmic vesicle part	258	11	29.57	.37	-	2.59E-04	3.43E-03

Gene Ontology enrichment analyses were conducted using the “Enrichment analysis” tool in the Gene Ontology website (http://geneontology.org/). Indentation represents the hierarchy of GO terms.
